# Stiffness of the Masseter Muscle in Children—Establishing the Reference Values in the Pediatric Population Using Shear-Wave Elastography

**DOI:** 10.3390/ijerph18189619

**Published:** 2021-09-13

**Authors:** Cyprian Olchowy, Anna Olchowy, Aleksander Pawluś, Mieszko Więckiewicz, Luca Maria Sconfienza

**Affiliations:** 1 Department of Oral Surgery, Wroclaw Medical University, 50-425 Wroclaw, Poland; 2 Department of Experimental Dentistry, Wroclaw Medical University, 50-425 Wroclaw, Poland; ania.olchowy@gmail.com (A.O.); m.wieckiewicz@onet.pl (M.W.); 3 Department of General Radiology, Provincial Specialist Hospital in Legnica, 59-220 Legnica, Poland; apawlus@wp.pl; 4 Unità Operativa di Radiologia Diagnostica e Interventistica, IRCCS Istituto Ortopedico Galeazzi, 20161 Milano, Italy; io@lucasconfienza.it; 5 Dipartimento di Scienze Biomediche per la Salute, Università degli Studi di Milano, 20161 Milano, Italy

**Keywords:** shear-wave elastography, masseter, temporomandibular disorders, elasticity, pediatric population

## Abstract

In children, the quality and muscle function are altered in many pathologic conditions, including temporomandibular disorders. Although several methods have been used to evaluate muscle tonus, none became a golden standard. Moreover, the masseter muscle characteristics in children have not been investigated to date. This study aimed to measure the stiffness of the masseter muscle using shear-wave elastography in healthy children. We enrolled 30 healthy children (mean age 10.87 ± 3.38 years). The stiffness of masseter muscles was measured with shear wave elastography. Stiffness for the total sample was 6.37 ± 0.77 kPa. A comparison of the measurements did not show significant differences between the right and the left masseter muscles (left—6.47 ± 0.78 kPa; right—6.24 ± 0.76 kPa; *p* = 0.3546). A significant difference was seen between boys and girls (boys—5.94 ± 0.50 kPa; girls—6.63 ± 0.80; *p* = 0.0006). Shear-wave elastography is a promising diagnostic tool. It may help to detect changes in the stiffness of the masseter muscle and draw attention to pathological processes within the jaw muscles. Directions for further research shall include determining stiffness values in pathological conditions and the impact of biological and functional factors on the stiffness of the masseter muscle.

## 1. Introduction

The quality and muscle function are altered in many pathologic conditions. In children, disturbed muscle functioning can be associated with hereditary diseases (muscular and neuromuscular dystrophies) [1], injuries to the brain resulting in cerebral palsy [2], juvenile idiopathic arthritis [3], locomotor system disabilities [4] and temporomandibular disorders (TMD) [5]. It has been estimated that atypical muscle tone affects about 5–8% of children [6]; however, local changes in the muscle tone may be more frequent.

TMD is an umbrella term that embraces chronic pain conditions and dysfunction in the orofacial region affecting the masticatory muscles, the temporomandibular joints and their associated structures. TMD are often associated with pain that can have muscle origin [7,8]. The prevalence of TMD in the general adult population in Poland is estimated to be as high as 55.9% [9]. Such data are not available in the population of Polish children and adolescents, but the available studies on other country populations indicate that TMD prevalence in children and adolescents range from 7.3% to 30.4% [10,11], although the prevalence of TMD in adolescents can be even as high as 33% as highlighted by the study conducted in Brazil [12]. The prevalence of TMD is believed to increase with age [13].

Methods of assessing muscle condition help monitor the progression of the disease and the evaluation of implemented treatment. Some attempts were reported on the use of myotonometry to evaluate muscle stiffness both in healthy people and children with Duchenne muscular dystrophy [14,15]. Surface electromyography, used for the measurement of muscle activity, can also help evaluate a muscle condition in children with cerebral palsy [16]. In addition, it was shown that in children with TMD, electromyography was able to detect the lower activity of the masseter muscle in comparison with children without abnormalities in this muscle [17]. It is worth noting that biomechanical methods lack standardization. They are characterized by high variability, which hampers comparisons between individuals and the conduct of clinical research [16]. In addition to biomechanical methods, several clinical scales are used (e.g., Ashworth Scale) [18] to assess muscle tone, but there is no agreement among clinicians on the gold standard. Finally, there is no method for the routine assessment of hypotonia [19].

Shear-wave elastography is gaining attention in recent years. This method was validated in studies with phantoms of known hardness and comparative studies [20,21]. The results of studies on internal organs such as the thyroid, kidneys or liver, show that the agreement between the radiologists is statistically significant [22,23,24]. There are also some attempts to use shear-wave elastography for measuring muscle stiffness. Goo et al. conducted a systematic review to identify methods used for the evaluation of skeletal muscle mechanical properties in the pediatric population [6]. They identified 60 studies with data published in 40 articles on the shear-wave elastography conducted with Aixplorer (Supersonic Imagine, France). They concluded that this method was characterized by strong (+++) intra-rater, inter-rater and test-retest reliability. In the literature, shear-wave elastography was used to measure spasticity in children with cerebral palsy [25], Duchenne muscular dystrophy [26] and muscle condition in scoliosis [27]. The examined muscles included gastrocnemius muscle [25], tibialis anterior [26], vastus lateralis [26], biceps brachii [26], triceps brachii [26], adductor digitorum minimi [26] and lateral abdominal muscle [27]. Another advantage of shear-wave elastography is its ability to identify trigger points and help to make a diagnosis of myofascial pain syndrome [28]. Among other advantages of shear-wave elastography, it is its simplicity that allows dentists and other specialists to monitor disease response to treatment [29].

The assessment of masticatory muscles would be useful in the treatment of TMD and other pathologies generally affecting muscle condition; however, the masseter muscle characteristics in children have not been investigated to date. For this reason, the aim of this study was to measure the stiffness of the masseter muscle using shear-wave elastography in healthy children.

## 2. Materials and Methods

This study included 30 healthy children, namely older than one year of age and younger than 18 years of age. We aimed to include healthy children in terms of the lack of any systemic and oral disease or pathologic condition. Subjects with symptoms or a history of any disease that affects muscle tonus, including neuromuscular disorders, cancer, symptoms of TMD, oral parafunctions and orofacial pain, were not considered. In addition, patients who were taking muscle relaxants, other drugs affecting the muscles, analgesics and botulin injections in the examined area were not included. Parents were asked for written informed consent for the participation of their child in the study. All children gave their consent for participation in the study as well. The study was approved by the Bioethical Committee at the Wroclaw Medical University (KB—633/2020). The study was conducted in accordance with the Declaration of Helsinki and Good Clinical Practice.

Stiffness of masseter muscles was measured with shear wave elastography using Aixplorer Ultimate device (SuperSonic Imagine, Aix-en-Provence, France) with a high-frequency linear probe SL 18-5 (5–18 MHz). All children were examined in a supine; they were instructed to lay down relaxed with their mouth closed in a comfortable position as well as to refrain from swallowing during the examination. A small amount of an ultrasound gel was used for better visualization. The probe was placed longitudinally to the long axis of the masseter muscle. The measurements were registered in the widest and thickest part of the muscle belly—half-length in between the origin and insertion of the masseter muscle [30,31]. The Region of Interest (ROI) diameter of 4 mm was selected. No pressure was applied by the probe of the muscle. Each muscle was scanned three times; hence, the mean was analyzed. All examinations were performed by a radiologist with seven years of experience in elastography. Measurements were validated using an elasticity QA Phantom model 049A (Computerized Imaging Reference Systems, Inc, Norfolk, VA, USA). Inter- and intraobserver agreements between the stiffness measurements of the masseter muscle performed using the Aixplorer Ultimate device were rated excellent and confirmed diagnostic accuracy of this method [29]; however, in this study, the calculation of the intraobserver agreement was not included.

Data were statistically analyzed with the R Project for Statistical Computing v. 3.4.1. The data were presented as means with standard deviations and medians with interquartile range (IQR). Normality was checked with the Shapiro–Wilk test; all variables except for left masseter stiffness measurement had distribution other than normal. Stiffness measurements were compared with the Wilcoxon rank-sum. Differences were considered statistically significant at *p* < 0.05. The sample size calculation was based on the following assumptions: type I error (α = 0.05), type II error (β = 0.10), the difference of means of 0.56, standard deviations of 0.65 and an equal number of measurements in both tested groups due to comparisons between sides of the body. Such assumptions set the minimum sample size of 30 participants.

## 3. Results

The study included 30 children (12 boys and 18 girls). The range of age for girls was from 1 to 15 years and for boys, it was from 9 to 14 years. The mean age was 10.87 ± 3.38 years, with girls not significantly younger than boys. Stiffness for the total sample was 6.37 ± 0.77 kPa. The detailed values of the masseter muscle stiffness are presented in Table 1.

Comparisons of the measurements did not show significant differences between the right and the left masseter muscles, which is shown in Table 2. However, differences could be observed between boys and girls.

**Figure 1:**
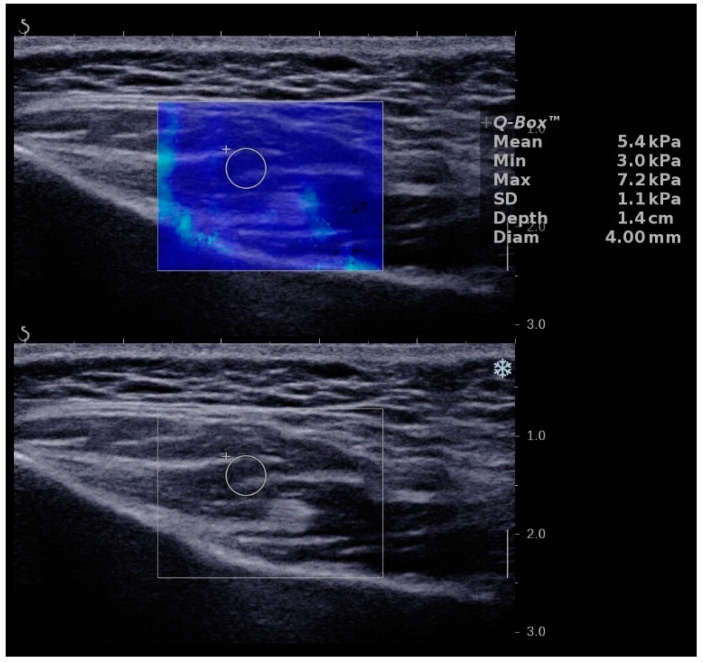
Elasticity of masseter muscle of a 12-year-old boy.

Figure 1 shows an exemplary measurement of elasticity of the masseter muscle of a 12-year-old boy in which the homogenous stiffness of the masseter muscle within the box (homogenous blue color) can be seen. This image is characteristic for underage people with a mean elasticity of 5.4 kPa.

## 4. Discussion

The present study showed that in healthy children, the stiffness of the masseter muscle measured with shear-wave elastography was 6.37 ± 0.77 kPa on average. Similar values for the right and left masseter muscle, but the values in girls were higher than those recorded in boys (boys—5.94 ± 0.50 kPa; girls—6.63 ± 0.80; *p* = 0.0006). This study provided the preliminary characteristics of the small sample and, thus, further research has to be conducted to investigate masseter muscle stiffness in healthy children, including the impact of age, sex and dentition type, as well as any pathologic conditions. For this reason, our results should be interpreted with caution.

Little is known about the stiffness of the masseter muscle in the pediatric population. Up to date, only one preliminary study reported masseter muscle stiffness in healthy children and adolescents. In this study, however, the mean stiffness value was very high and amounted to 16.96 ± 9.01 kPa with the closed mouth [32]. Because there is no other evidence on the stiffness of the masseter muscle, we show here that every muscle has its own stiffness characteristics and requires individual reference values. Several studies examined stiffness of the gastrocnemius lateralis muscle in children with typical development. Brandenburg et al. showed that stiffness values depended on a degree of plantar flexion and ranged from 7.8 kPa (IQR: 6.1, 11.0) for 20 degrees to 14.9 kPa (IQR: 10.9, 20.9) when the foot was in a natural position without any flexion. Their study included 13 children with a median age of median age 5 years and 3 months [25]. Lacourpaille et al. measured stiffness for six muscles and recorded the results at two muscle lengths (shortened and stretched). In their study, the mean age of the healthy control group (*n* = 13) was 12.86 ± 5.5 years. The following mean values for two muscle positions were given: for the tibialis anterior muscle—7.0 ± 1.9 kPa and 12.5 ± 3.1 kPa; for the gastrocnemius medialis muscle—4.9 ± 0.9 kPa and 14.5 ± 3.4 kPa; for the vastus lateralis muscle—5.4 ± 1.7 kPa and 9.6 ± 2.2 kPa; for the biceps brachii—3.9 ± 0.4 kPa and 18.9 ± 6.4 kPa; for the triceps brachii—5.0 ± 0.9 kPa and 7.3 ± 1.4 kPa; and for the abductor digiti minimi—7.5 ± 2.2 kPa and 11.9 ± 5.0 kPa [33]. Another study by Lacourpaille et al. showed that values of muscle stiffness measured in 5 muscles were comparable over a 12-month follow-up in the control group (*n* = 9) but differed for some muscles in children with Duchenne muscular dystrophy (*n* = 10) [26]. The assessment of lateral abdominal muscles in children with scoliosis was carried out by Linek et al. [27]. In a supine rest position, the following values were reported: for the external oblique muscle—21.1 ± 9.19 kPa on the right side and 18.8 ± 7.34 kPa on the left side; for the internal oblique muscle—14.6 ± 4.24 kPa on the right side and 13.4 ± 4.54 kPa; and for the transversus abdominis muscle—13.8 ± 4.22 kPa on the right side and 11.5 ± 3.76 kPa on the left side. The study group included 35 children aged 12.8 ± 2.8 years on average. Although the groups in those studies were small, it can be observed that each muscle has its unique range of stiffness values. For this reason, there is a need to characterize each of the muscles separately and provide values for the healthy muscle and each pathological condition individually. There is also a need to investigate factors influencing muscle stiffness such as age, treatments or exercise because such factors were proved to change masseter muscle stiffness in adults [34,35].

A systematic review by Olchowy et al. showed that stiffness depends not only on the type of the muscle but also on the device that was used for measurements [36]. Regarding the masseter muscle, several studies reported stiffness of this muscle; however, all were conducted in the adult cohorts. Three of the studies used the Aixplorer device, the same as we did for our measurements. Arda et al. examined 127 healthy volunteers; the average stiffness of the masseter muscle in this group was 10.4 ± 3.7 kPa [37]. In the study conducted by Herman et al., the mean stiffness of the masseter muscle in 176 healthy volunteers was 10.0± 4.3 kPa [38]. The study by Olchowy et al. conducted on 140 healthy adults determined the elasticity to be 10.67 ± 1.77 kPa [39]. The average value obtained in the pediatric population in the present study was 6.37 ± 0.77 kPa, which was about one third less than in adults. The reason for higher stiffness in adults is the fact that stiffness increases with age due to muscular atrophy and a change in fibre composition and distribution and the muscle-tendon complex [40,41]. We can therefore conclude that stiffness of the masseter muscle in children is lower than in adults. Chodock et al. using the Aixplorer ultrasound device found that age was a significant predictor of a mean shear-wave velocity only for the muscles of the sternocostal region of the pectoralis major and the middle trapezius, but not for the anterior deltoid, biceps brachii and the muscles of the clavicular region [42]. Differences in muscle tonus were also confirmed by other methods such as myotonometry. Dietsch et al. investigated tissue stiffness using myotonometry in 40 healthy adults aged from 18 to 90 years old. They found that values of stiffness were significantly higher in older participants aged from 61 to 90 years old in comparison to younger ones aged from 22 to 37 years old [14]. In contrast, Sendur et al. found that stiffness of the gastrocnemius muscle decreased with age in a group of 57 healthy participants aged 18 to 74 years [43]. These conflicting results add another reason for the need to characterise each muscle separately. Olchowy et al. reported that stiffness increased after a massage session [34].

The muscle stiffness difference described between children and adults is especially interesting in the context of the prevalence of muscle disorders, particularly masticatory muscles disorders. As mentioned in the introduction, TMD seems to increase with age and thus affect adults more frequently than children and adolescents. While the prevalence of TMD in adults is reported to affect 55.9% [9], the highest prevalence of TMD reported in adolescents was 33% [12]. As reported in the previous studies, the stiffness of masseter muscles seems to be higher in individuals presenting masticatory myofascial pain than in healthy controls. As also previously mentioned, general muscle stiffness was reported to increase with age [44] and one of the most common TMD symptoms is increased muscle tension and pain [45]. This information leads to the hypothesis that lower basial stiffness of masticatory muscles in children and adolescents can be possibly associated with higher masticatory muscles adaptation ability and the lower muscle-origin TMD prevalence. However, this topic requires further research.

## 5. Conclusions

Shear-wave elastography is a promising diagnostic tool. It may help to detect changes in stiffness of the masseter muscle and draws attention to pathological processes within the jaw muscles. Directions for further research shall include further determination of stiffness values in pathological conditions and the impact of biological and functional factors on the stiffness of the masseter muscle.

## Figures and Tables

**Table 1:** Age and results values of stiffness measured by shear-wave elastography.

Group	Mean ± SD	Median (IQR)
Total		
Age (year)	10.87 ± 3.38	12.00 (9.25–13.00)
Left masseter (kPa)	6.47 ± 0.78	6.33 (6.11–7.04)
SD of left masseter	1.45 ± 0.42	1.41 (1.11–1.63)
Right masseter (kPa)	6.24 ± 0.76	6.30 (5.58–6.71)
SD of right masseter	1.41 ± 0.52	1.40 (0.91–1.83)
Girls		
Age (year)	10.28 ± 4.16	11.50 (8.25–13.00)
Total value (kPa)	6.63 ± 0.80	6.68 (6.10–7.13)
Left masseter (kPa)	6.72 ± 0.85	6.87 (6.17–7.13)
SD of left masseter	1.62 ± 0.42	1.60 (1.37–1.70)
Right masseter (kPa)	6.54 ± 0.77	6.63 (5.87–6.77)
SD of right masseter	1.61 ± 0.53	1.73 (1.00–2.03)
Boys		
Age (year)	11.75 ± 1.42	12.00 (11.00–13.00)
Total value (kPa)	5.94 ± 0.50	6.12 (5.55–6.30)
Left masseter (kPa)	6.09 ± 0.46	6.17 (6.10–6.31)
SD of left masseter	1.20 ± 0.30	1.12 (1.06–1.31)
Right masseter (kPa)	5.80 ± 0.52	5.67 (5.50–6.30)
SD of right masseter	1.10 ± 0.33	0.92 (0.86–1.40)

**Table 2:** Comparison between groups.

Comparison	*p*-Value
Age boys/age girls (year)	0.5354
Left masseter total/right masseter total	0.3546
Masseters boys/masseters girls	0.0006
Left masseter girls/right masseter girls	0.3420
Left masseter boys/right masseter boys	0.2973
Left masseter girls/left masseters boys	0.0416
Right masseter girls/right masseters boys	0.0030

## Data Availability

The data presented in this study are openly available in FigShare at doi:10.6084/m9.figshare.16608619.
